# Mechanisms of Remodeling in Asthmatic Airways

**DOI:** 10.1155/2012/316049

**Published:** 2012-01-19

**Authors:** Adrian Shifren, Chad Witt, Chandrika Christie, Mario Castro

**Affiliations:** Division of Pulmonary and Critical Care Medicine, Washington University School of Medicine, St. Louis, MO 63110, USA

## Abstract

Asthma is a chronic inflammatory airway disorder characterized by airway hyperresponsiveness and reversible airflow obstruction. Subgroups of asthma patients develop airflow obstruction that is irreversible or only partially reversible and experience an accelerated rate of lung function decline. The structural changes in the airways of these patients are referred to as airway remodeling. All elements of the airway wall are involved, and remodeled airway wall thickness is substantially increased compared to normal control airways. Airway remodeling is thought to contribute to the subphenotypes of irreversible airflow obstruction and airway hyperresponsiveness, and it has been associated with increased disease severity. Reversal of remodeling is therefore of paramount therapeutic importance, and mechanisms responsible for airway remodeling are feasible therapeutic targets for asthma treatment. This paper will focus on our current understanding of the mechanisms of airway remodeling in asthma and potential targets for future intervention.

## 1. Introduction

Asthma is a chronic inflammatory disorder of the airways characterized by airway hyperresponsiveness (AHR) and reversible airflow obstruction that fluctuates over time. Asthma used to be considered a single disease entity, but is increasingly recognized as a disease with multiple subphenotypes that differ in clinical severity, pathological findings, response to therapy, and long-term outcome [1]. A subgroup of this heterogeneous group of asthma patients manifests airflow obstruction that is either irreversible or only partially reversible. Furthermore, some of these patients experience an accelerated rate of decline in respiratory function compared to healthy controls [2, 3]. In children with asthma defined by wheezing diagnosed at age 7 and followed for 21 years, lung function was essentially normal in patients who ceased wheezing but was increasingly abnormal in those patients who continued to wheeze frequently throughout life [4].

Airway inflammation, tissue injury, and subsequent abnormal repair lead to structural changes in the airway walls of asthmatic subjects collectively referred to as airway remodeling. Airway remodeling is strongly suspected to result in the physiologic subphenotypes of irreversible or partially reversible airflow obstruction and accelerated lung function decline [5]. Almost all elements of the airway wall have been shown to be altered in fatal asthma [6, 7]. The changes occur throughout the bronchial tree [8], but are most marked in large membranous and small cartilaginous airways [9]. Similar findings occur in nonfatal asthma, although they are less profound and localized predominantly to midsized and small membranous airways less than 3 mm in diameter [6, 10]. This review will focus on our current understanding of the pathology, pathogenesis, and physiologic consequences of airway remodeling in asthma, and discuss potential targets for therapeutic intervention.

## 2. Pathology and Pathogenesis of Airway Remodeling

### 2.1. Airway Wall Thickening

In fatal asthma, airway wall thickness is increased by between 50 and 230% compared to normal controls, while in nonfatal asthma, the increase ranges from 25–150% [11]. Increased wall thickening has repeatedly been associated with increased disease severity, including near fatal asthma [12–14]. These changes are the result of epithelial cell alterations, subepithelial fibrosis, submucosal gland hyperplasia, increased airway smooth muscle mass, and increased airway vascularization [10, 15–17].

Evaluation of airway wall thickening by multidetector-row computed tomography (MDCT) is a promising noninvasive technique for assessing airway remodeling [18]. Quantitative MDCT imaging allows precise measurement of airway wall area (WA) and airway wall thickness (WT) out to sixth-generation bronchi. Several studies have compared pathologic changes of airway remodeling with increased wall thickness as measured by MDCT. Both WA and WT percentages have been shown to correlate with histologic basement membrane thickening [19] and moderate correlations exist between WA and WT percentage and epithelial layer thickness [20]. In addition, quantitative CT measures, such as WA and WT, at multiple airway generations appear to correlate with FEV_1_ and bronchodilator responsiveness [20]. Thus, quantitative CT scans, as a surrogate noninvasive measure of remodeling of the airways, may be used as an endpoint for targeted therapy to reverse airway remodeling or to potentially predict those individuals at risk of progressive remodeling; however, further evaluation of this modality is needed.

### 2.2. Allergic Airway Inflammation

**Figure 1:**
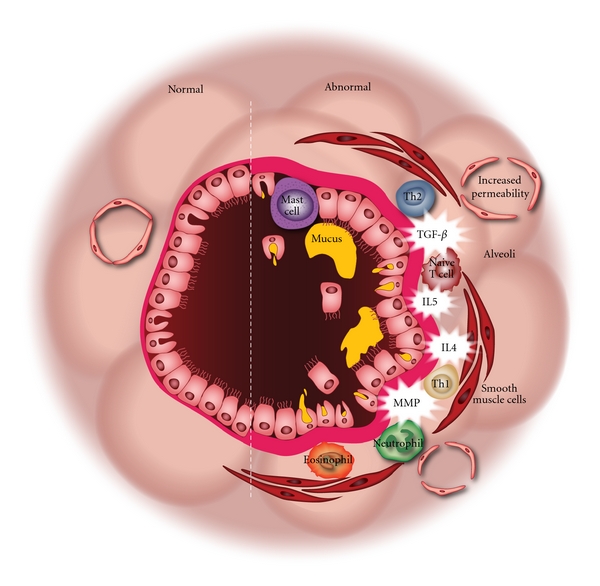
Airway remodeling (abnormal half of figure) involves almost all elements of the airway wall and occurs throughout the bronchial tree. Although atopy-related inflammation is considered the primary cause of asthmatic airway remodeling, insults such as tobacco smoke and viral pathogens induce a similar histologic phenotype.

Chronic inflammation that results in tissue injury with subsequent structural change during tissue repair is a well-documented biological phenomenon, for example, cirrhosis. Since chronic airway inflammation is a striking feature of asthma, asthmatic airway inflammation is often assumed to be the initiating event for airway remodeling [21]. Most, but not all, asthma is associated with atopy, and as such, asthma has largely been regarded as an allergic disease [22]. In keeping with this premise, a cellular infiltrate rich in lymphocytes, eosinophils, mast cells, neutrophils, and macrophages [5] characterizes asthmatic airway inflammation. Lymphocytic inflammation is dominated by Th2-cells producing interleukin (IL)-4, IL-5, and IL-13 (Figure 1) [23]. Overexpression of Th2 interleukins in mouse models has demonstrated changes pathognomic of asthmatic airway remodeling. Overexpression of IL-13 resulted in subepithelial fibrosis, mucus metaplasia, and an inflammatory infiltrate rich in eosinophils and macrophages [24]. IL-5 overexpression induced striking airway eosinophilia, along with mucus metaplasia and subepithelial fibrosis [25]. While IL-4 overexpression led to eosinophilia and mucus metaplasia, subepithelial fibrosis in IL-4 overexpressing models has been unimpressive or absent depending on the study [26, 27]. In addition, mice overexpressing all three of these Th2 interleukin molecules demonstrated AHR. Eosinophils and mast cells likely impact the epithelial remodeling based on effects on barrier function, epithelial proliferation and desquamation, and goblet cell formation [28]. These data indicate that airway remodeling is quite likely driven in part by the Th2 inflammation characteristic of the asthmatic airway. 

Several lines of evidence in animal models and humans suggest that the Th2 hypothesis is an incomplete explanation for asthma pathogenesis. First, many patients with asthma do not have an identifiable allergic trigger for their disease. Conversely, a significant number of patients with atopy do not develop asthma [29]. Taken together, it is reasonable to deduce that other triggers exist for generating asthma. Second, the allergic and nonallergic forms of asthma are pathologically indistinguishable from each other [30], implying that remodeling occurs independent of atopic inflammation. This suggests a remodeling pathway common to all forms of remodeled asthma. Third, current anti-inflammatory and allergen-reduction therapies (see below) do not prevent the development of asthma or effectively reverse airway remodeling once it has occurred [31]. Lastly, airway remodeling occurs simultaneously with inflammation and may indeed be necessary for the establishment of a chronic inflammatory state [32]. These observations have encouraged exploration of alternative mechanisms of airway injury as the underlying mechanism of airway remodeling.

### 2.3. Epithelial-Driven Models of Airway Remodeling

While it is likely that some aspects of airway remodeling are the end result of allergen exposure and subsequent chronic allergic inflammation, it is increasingly believed that predisposition to asthma lies in a structurally and functionally defective airway epithelium which links the inhaled environment to underlying airway structures. This phenomenon is best explained by the model of the epithelial mesenchymal trophic unit or EMTU proposed by Holgate [[33] and reviewed in [34, 35]]. In this model, both airway inflammation and remodeling are the consequence of repetitive environmental injury to a defective airway epithelium by viruses, air pollutants, or tobacco smoke (Figure 1) [22]. Injury leads to interaction between the dysfunctional epithelium and the underlying mesenchyme that results in amplification of the inflammatory and remodeling responses in the underlying layers of the airway wall with subsequently defective airway repair [35].

Support for this model is increasingly robust. Evidence, primarily from animal models, indicates that innate immune responses to respiratory virus infections, for example, contribute to the development of inflammatory airway disease characteristic of asthma [36–39]. Paramyxoviral infection of mice has been shown to produce acute bronchiolitis resulting in airway inflammation and AHR. However, infection also results in a chronic inflammatory response with airway remodeling and AHR phenotypes [40]. This chronic response is not only strikingly similar to the inflammatory response in the airways of asthmatic patients, but also persists for over a year after mice have cleared the virus from the airways. It has subsequently been shown that the chronic inflammatory state is related to the severity of infection and is produced by an innate epithelial immune response in which natural killer T cells activate macrophages to produce proinflammatory cytokines like IL-13, which contribute to chronic mucous cell metaplasia [[41] and are reviewed in [42, 43]]. The mouse model correlates well with clinical findings. Paramyxoviral infections are a primary cause of lower respiratory tract infection in infants and children [44], and children with severe RSV bronchiolitis are predisposed to development of a chronic wheezing illness in the absence of both atopy and viral persistence in airway tissue [45, 46].

### 2.4. Epithelial Cell Alterations

**Figure 2:**
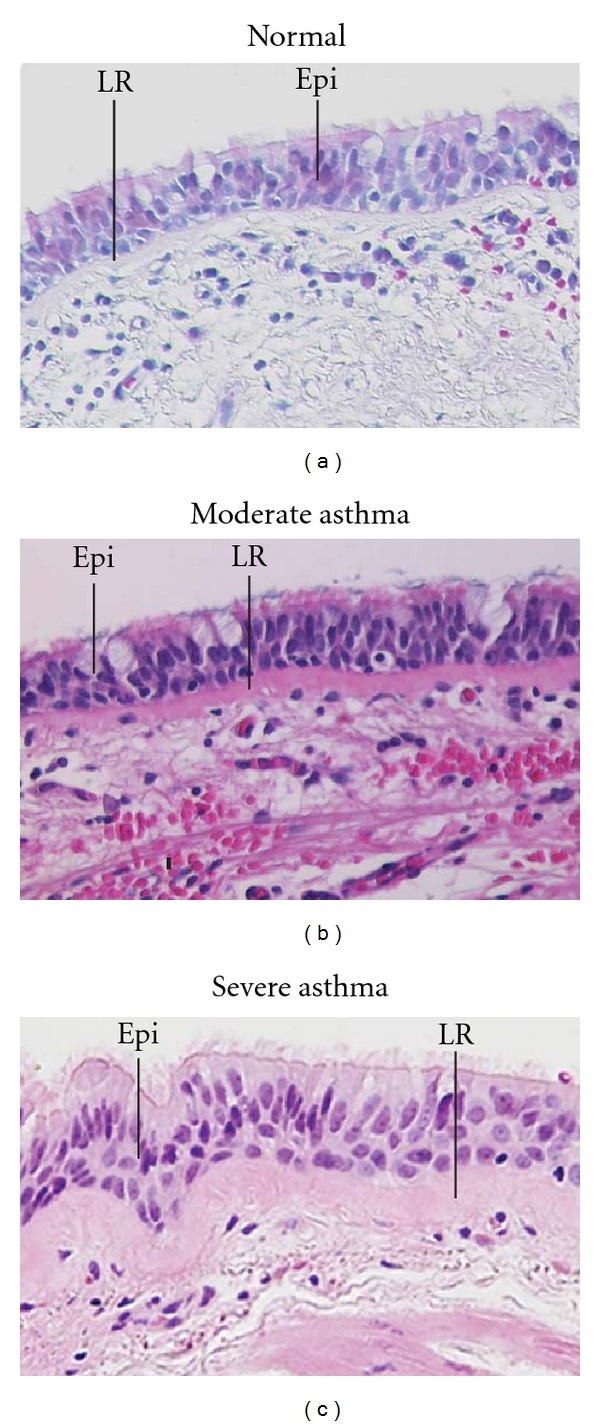
Hematoxylin and eosin stained endobronchial biopsies from control, moderate asthmatic, and severe asthmatic patients. Lamina reticularis (LR) and epithelium (Epi) are labeled. Note the increased thickness of both the LR and epithelium as asthma severity increases. Mag = 20x.

**Figure 3:**
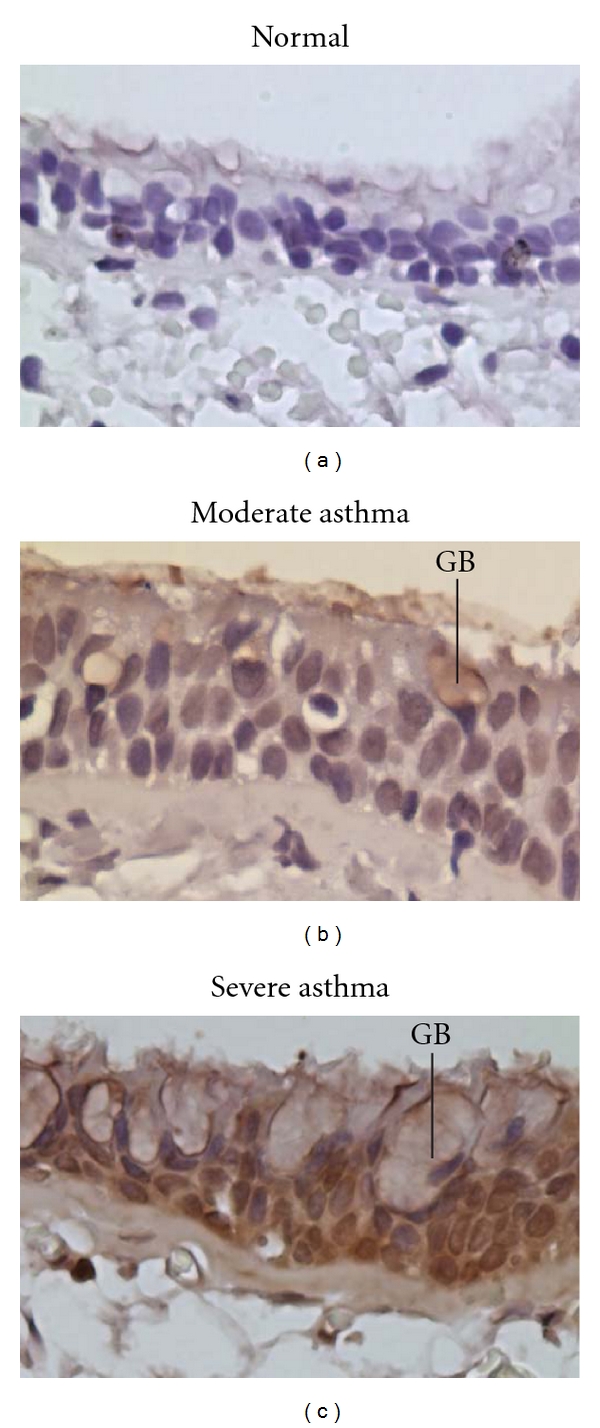
Endobronchial biopsy specimens from control, moderate asthmatic, and severe asthmatic patients stained with antiretinoblastoma (anti-Rb) antibody, a marker of cell proliferation. Rb-positive cells stain brown. There is a significant increase in Rb-positive epithelial cells as asthma severity increases. There is also an increase in the number and size of goblet cells (GB) in the epithelial layer as asthma severity increases. Mag = 40x.

Epithelial cell shedding, ciliated cell loss, and goblet cell hyperplasia have all been described in asthmatic airways [6, 15, 47]. Epithelial shedding has been noted in postmortem studies of asthmatic airways, and sputum and BAL samples from asthmatic patients contain increased amounts of epithelial cells [6, 48]. However, epithelial cell desquamation in bronchial biopsy specimens from healthy nonasthmatic subjects appears similar to that seen in biopsies from mild to severe asthmatics [49, 50] suggesting that this phenomenon is related to the sampling technique itself. Evidence of increased epithelial cell proliferation contributing to thickening of the epithelium and an increased lamina reticularis (also known as subepithelial fibrosis, see below) has been observed in patients with moderate to severe asthma (Figure 2) while being absent in patients with mild persistent asthma, chronic bronchitis, and normal controls [49]. These studies suggest that thickening of the airway seen in severe asthma may be due, in part, to airway epithelial proliferation (Figure 3), although conflicting data exist (see below). 

Goblet cell hyperplasia has been consistently demonstrated in mild, moderate, and severe forms of asthma (Figure 3), although the finding is particularly apparently increased in severe and fatal asthma [51, 52]. Similarly, an increase in the area of airway wall occupied by submucosal mucus glands is a frequent finding in asthmatic airways, and occurs in both fatal and nonfatal forms of asthma [6]. Goblet cells produce mucin glycoproteins (MUC), 13 of which have been identified in human airways [53]. The dominant mucin in humans is MUC5AC, which is expressed in the airways of normal subjects and is upregulated in asthmatic subjects [54]. Goblet cell hyperplasia has been demonstrated following adoptive transfer of Th2 cells into ovalbumin-challenged mice [55]. This is most likely the result of Th2-driven interleukin expression (see above). IL-13, in particular, signals through the STAT-6 signaling pathway [5] and the effects of IL-13 overexpression in mice are almost completely STAT-6 dependent [56].

Other changes observed in the airway epithelium lend support to the EMTU hypothesis of asthma pathogenesis. Epithelial injury is normally followed by upregulation of proteins responsible for tissue repair. Expression of epithelial growth factor receptor (EGFR) and MUC5AC are both markedly upregulated in the epithelium of asthmatic patients [57, 58], and have been shown to colocalize in goblet cells [59]. Immunoreactivity to EGFR and the total area of MUC5AC staining show a positive correlation in both asthmatics and control subjects. Furthermore, activation of EGFR has been shown to upregulate both mucin production and goblet cell generation in human epithelial cells *in vitro* [57]. Interestingly, increased airway expression levels of EGFR are not associated with markers of increased cell proliferation as would be expected in tissue undergoing active repair [60] suggesting an innate defect of the asthmatic epithelium to repair itself.

### 2.5. Subepithelial Fibrosis

The original report of airway remodeling described the phenomenon of basement membrane thickening [61]. Electron microscopy has subsequently shown that thickening occurs just below the true basement membrane in a zone known as the lamina reticularis [17]. The lamina reticularis (Figure 2) is a collagenous layer 4-5 *μ*m thick in control subjects. In asthmatics, lamina reticularis thickness has been documented at between 7 and 23 *μ*m [62]. Thickening is the result of extracellular matrix deposition, primarily collagens I, III, and V [5]. In addition, abnormalities of noncollagenous matrix, including elastin, fibronectin, tenascin, lumican, and proteoglycans, have also been described [17, 63, 64].

Subepithelial fibrosis occurs in children, and is similar in extent to that seen in adults [65] suggesting that it is an early finding of asthmatic airway remodeling. Subepithelial fibrosis has also been reported in all severities of asthma [9, 66]. However, the specificity of subepithelial fibrosis is called into question by studies that have identified severe asthmatics without subepithelial fibrosis, and nonasthmatic subjects with significant fibrosis [67–69]. Furthermore several functional measurements of asthma show variable correlations with the degree of fibrosis [66, 70–72] raising questions about its functional consequences.

Myofibroblasts are probably key effectors of subepithelial fibrosis. Myofibroblasts are specialized cells with phenotypic characteristics of both fibroblasts and myocytes [53]. They express *α*-smooth muscle actin, produce inflammatory mediators, and are major producers of extracellular matrix proteins necessary for tissue repair and remodeling.

Transforming growth factor- (TGF-) *β* mediates the effects of IL-13 overexpressing mice [73]. TGF-*β* is a cytokine produced by multiple lung cells including epithelial cells, macrophages, fibroblasts, lymphocytes, and eosinophils [53]. TGF-*β* induces fibroblasts to express *α*-smooth muscle actin and assume a myofibroblast phenotype [74]. As part of normal wound repair, TGF-*β* induces expression and secretion of multiple extracellular matrix proteins while also inhibiting their degradation. In many diseases, excessive TGF-*β* results in an excess of pathologic tissue fibrosis leading to compromised organ function [75]. Compared to controls, TGF-*β* expression is increased in asthmatic airways and BAL fluid. In addition, TGF-*β* levels correlate with the extent of subepithelial fibrosis, airway fibroblast numbers, and disease severity [76–78]. Thus, excess TGF-*β* production may be pivotal for the development of subepithelial fibrosis.

Matrix metalloproteinases are zinc-dependent endopeptidases capable of degrading extracellular matrix molecules. The dynamic equilibrium between matrix metalloproteinases and their inhibitors is a critical determinant of matrix remodeling [79]. The existence of increased subepithelial fibrosis in asthmatic airways strongly suggests that a profibrotic balance exists between the two. In asthma, the most important metalloproteinase molecules are MMP-9 and its inhibitor, tissue inhibitor of metalloproteinase- (TIMP-) 1 [5]. Both MMP-9 and TIMP-1 levels are elevated in airway biopsies and BAL fluid of asthmatic patients [80–82]. However, compared to control subjects, asthmatics have a significantly lower MMP-9 to TIMP-1 ratio supporting a profibrotic balance (inhibition over degradation). In addition, the lower MMP-9 to TIMP-1 ratios correlate with the degree of airway obstruction [83].

TGF-*β* is secreted from cells as a latent complex and is targeted to the extracellular matrix by latent TGF-*β* binding proteins for subsequent activation [84]. MMPs regulate matrix-bound cytokine release [83], and activation of TGF-*β* is MMP-9 dependent [73]. Therefore, the role of elevated levels of MMP-9 in asthma may be related to TGF-*β* activation and its downstream fibrotic sequelae [5].

Thickening of the lamina reticularis provides further evidence for the idea of a dynamically interactive EMTU. Epithelial disturbances have been shown to result in increased levels of fibrogenic growth factors including both the latent and active forms of TGF-*β* [33, 85]. This response is enhanced in asthmatic epithelium compared to normal controls, and the fibrogenic factors have been shown to localize in mesenchymal elements underlying the injured epithelium [86, 87] including the lamina reticularis. Thus, the lamina reticularis may act as a conduit for transmission of signals from an innately defective epithelium to deeper tissues of the airway wall.

### 2.6. Increased Airway Smooth Muscle Mass

Increased airway smooth muscle (ASM) mass is the most prominent feature of airway remodeling [6], with ASM mass increasing disproportionately compared to the increase in total wall thickness [53]. It has been documented in both fatal and nonfatal asthma [11], and correlates with both disease severity and duration, being greater in fatal than nonfatal cases of asthma [6, 7, 10] and greater in older patients with fatal asthma than younger patients with fatal disease.

The increase in ASM mass may be the coordinated result of increased myocyte size (hypertrophy), increased myocyte number (hyperplasia), and potentially differentiation and migration of mesenchymal cells to ASM bundles [88–91]. Controversy exists regarding the relative contributions of hypertrophy and hyperplasia to ASM mass increases. The evidence for hyperplasia is relatively convincing [90, 92]. However, support for hypertrophy is conflicting, in part because documentation of increased cell size (width) may be subject to artifact resulting from cell shortening [32]. While studies have documented hypertrophy of ASM in severe asthma, particularly in smaller airways, other studies found no evidence for ASM hypertrophy in mild-moderate asthma [32].

Mitogens are chemical compounds that stimulate cell division and trigger mitosis. Mitogens play an integral role in the development of increased ASM mass typical of asthmatic airways. Mitogens bind receptor tyrosine kinases (RTK), G protein-coupled receptors (GPCR), and cytokine receptors. These receptor systems are all capable of producing increases in ASM mass in cell culture models [53]. The list of mitogens is extensive, and includes TGF-*β*, IL-1*β*, IL-6, thromboxanes, leukotrienes, histamine, tryptase, serotonin, vascular endothelial growth factor (VEGF), and numerous others [89, 93, 94]. The receptor systems regulate mitogenesis primarily through the phosphoinositide 3′-kinase (PI3K) and extracellular signal-regulated kinase (ERK) signaling pathways [95, 96]. The PI3K and ERK pathways activate transcription factors which phosphorylate D-type cyclins facilitating cell cycle progression [53]. Almost all of these mitogens have been identified in airway biopsies and BAL fluid from asthmatic patients or are detected in asthmatic airway cell cultures [21]. They may therefore represent targets for modulation of airway smooth muscle in asthmatic disease.

ASM cells are often noted in close proximity to the airway epithelium. This epithelial-muscle distance was measured at 67 *μ*m in asthmatics compared to 135 *μ*m in controls [70]. It has been postulated that mesenchymal airway cells differentiate into ASM with subsequent migration of the new ASM cells into muscle bundles [97]. Whether these phenomena occur *in vivo* is unknown, but reports indicate that cultured human ASM cells migrate in response to mitogenic stimuli [98]. Many of the mitogens involved in cell proliferation also induce ASM cell migration including TGF-*β*, IL-1*β*, and VEGF [21, 53].

### 2.7. Bronchial Neovascularization

Increased vascularity is frequently associated with chronic inflammation, and increased airway vascularity is well documented in asthma [16, 99]. Compared to controls, bronchial biopsies from asthmatic patients demonstrate an increase in the number and cross-sectional area of blood vessels, predominantly capillaries and venules, especially in the lamina propria [98, 100, 101]. It has been suggested that neovascularization worsens disease through increased vascular congestion, airway edema and inflammation, and global wall thickening [5]. In support of this idea, increases in airway vessel number have been shown to correlate with both disease severity and AHR [100, 102, 103].

VEGF is an angiogenic growth factor. It is a mitogen for vascular endothelial cells inducing endothelial cell proliferation and migration while inhibiting apoptosis. VEGF is an important factor in diseases associated with abnormal angiogenesis and wound repair [104]. Overexpression of VEGF in mice induces marked airway angiogenesis along with significant airway edema [105]. Interestingly, VEGF overexpressing mice also demonstrate increased Th2 cytokine expression, including IL-13 and TGF-*β*, and have evidence of subepithelial fibrosis and increased ASM mass. Furthermore, IL-13 overexpression is associated with increased levels of VEGF suggesting that a positive feedback loop promoting Th2 polarization may exist in asthmatic airways [106]. VEGF levels in sputum and BAL fluid from asthmatics are significantly increased and appear to correlate with disease activity [98, 107, 108].

## 3. Physiologic Consequences of Airway Remodeling

Functional data increasingly support the idea that remodeling-induced structural changes contribute to the subphenotypes of AHR and irreversible or partially reversible airflow obstruction [109, 110]. There are many potential etiologic mechanisms that link altered airway anatomy and asthmatic pathophysiology, but most are beyond the scope of this review.

The role of small airways in producing airflow obstruction and AHR seems to be greater than that of larger airways [111, 112]. Peripheral lung resistance is increased in all severities of asthma, even mild cases [113, 114]. Heterogeneity of peripheral bronchoconstriction is a major determinant of airflow obstruction, creating large increases in the load against which patients must breath [115, 116]. Although heterogeneous bronchoconstriction is well documented in wild-type animals and nonasthmatic human subjects [117–119], mathematical models indicate that heterogeneity of peripheral bronchoconstriction is significantly increased in asthmatic patients when compared to normal controls [120, 121].

Increased ASM mass is thought to be the most likely cause of AHR [122]. Asthmatic ASM exhibits increased contractility [123, 124], a finding initially ascribed to increased total ASM mass [7, 8, 10]. However, results from studies comparing ASM force generation in asthmatic ASM and normal controls are contradictory [125–127], in large part because of the difficulty in normalizing measured force to ASM mass [128]. A more likely explanation of AHR in asthmatic patients is an increase in the maximal velocity of shortening (V_max_) of ASM cells [128, 129]. Experimental evidence supporting an increase in V_max_ includes data from human [124, 130] and animal models [131]. 

Noncontractile elements of airway remodeling also contribute to AHR [109]. Increased airway extracellular matrix is one noncontractile element that may contribute to AHR. Decreased airway compliance is well documented in asthmatic patients [132, 133] and has been found to correlate inversely, although weakly, with increases in subepithelial fibrosis [134]. Therefore, increased extracellular matrix content may contribute to nonreversible airway obstruction by reducing airway distensibility. It has also been postulated that increases in extracellular matrix lead to an excess of matrix-bound cytokines and retention of soluble inflammatory mediators [135]. This results in worsening airway inflammation with subsequent chronic persistence of established AHR.

Finally, contractile and noncontractile elements of the remodeled airway wall may interact to increase airflow obstruction and AHR. Application of cyclic stresses to airway segments reduces ASM contractility [136, 137]. Decreased airway distensibility may reduce the cyclic stresses transmitted to ASM during breathing, reducing the cyclical stretching of ASM [138, 139]. ASM adapts to this attenuated stimulus by assuming a shorter resting length while retaining its ability to generate force. This mechanical plasticity, as the phenomenon is known, is an important feature of ASM biology affecting its contractile function [140]. The shortened muscle fibers enhance the airways predisposition to undergo excessive constriction during stimulation [110].

## 4. Therapeutic Targets for Airway Remodeling

The natural history of airway remodeling is poorly understood [9]. While the physiologic subphenotypes are more obvious in older patients with more severe disease of longer duration [141], airway remodeling is known to occur early on in the disease course [32]. Clinical trials of therapeutic intervention to prevent airway remodeling are currently lacking. Reversal of existing remodeling is therefore an important therapeutic objective since remodeling may often be present even at the time of asthma diagnosis.

### 4.1. “Anti-Inflammatory” Therapy

Animal studies of allergen-challenged models suggest that airway remodeling can be prevented, but also suggest that it cannot be fully reversed once initiated [142]. In general, therapies aimed at immunomodulation have proved disappointing. These include therapies directed against T cells (azathioprine, cyclosporine, and methotrexate) and Th-2 cytokine blockade (IL-4, IL-5 (see below) and IL-13) [143]. Some positive clinical data has been obtained, specifically with the use of corticosteroids, but in general, the data are mixed. In one study, treatment of asthmatic patients with inhaled corticosteroids (ICS) for 1 year demonstrated reductions in both subepithelial fibrosis and AHR [144]. The authors attributed the decrease in AHR to both reduced airway remodeling and decreased airway inflammation. However, other studies have shown mixed results. While they have demonstrated significant reductions in AHR, these same studies demonstrated either lack of airway remodeling after 8 weeks of ICS therapy [145], or no change in lung function (specifically postbronchodilator FEV_1_% predicted) when compared to placebo, despite treatment with ICS for 4 to 6 years [146].

### 4.2. Targeted Immunotherapy

Airway eosinophils are a key component of Th2 inflammation and are thought to be key effectors of both chronic inflammation and airway remodeling in asthma [21]. IL-5 is a key mediator of eosinophil activation and results in increase in the number of circulating, airway, and sputum eosinophils in mice [147]. Initial trials with mepolizumab, an anti-IL-5 monoclonal antibody, were disappointing. Although circulating eosinophils were dramatically reduced, airway eosinophils were only depleted by approximately 55% and there was no observable effect on circulating T cells [148, 149]. In addition, no significant effect on asthma outcomes or AHR were noted [149–151]. Subsequent trials of mepolizumab in severe asthma patients with persistent sputum eosinophilia have demonstrated a significant reduction in airway wall thickness measured by quantitative CT scanning (RB1 WA%/body surface area). In addition, significant reductions in the levels of circulating and sputum eosinophils were also noted when compared to placebo [152, 153]. However, no effects on AHR were identified.

Treatment with omalizumab, an anti-human IgE monoclonal antibody, has well-documented efficacy in improving asthma outcomes in a subgroup of patients with moderate to severe persistent asthma [154–159]. Patients in this subgroup demonstrate a confirmed atopic component and remain uncontrolled despite high-dose inhaled corticosteroids and at least one additional controller therapy [160]. Omalizumab inhibits the binding of IgE to the high-affinity IgE receptor (Fc*ε*RI). It does so by binding to an epitope on the IgE molecule to which the Fc*ε*RI would bind [161]. Omalizumab resulted in significant reductions in sputum eosinophil counts and an 80% reduction in the number of airway tissue eosinophils. Even more striking was an almost complete reduction in airway cells staining positive for Fc*ε*RI (including basophils and mast cells) and a significant decrease in the number of airway T and B cells [162].

The therapeutic utility of anti-IgE therapy stands in stark juxtaposition to that of anti-IL-5 therapy. The differences suggest that either the (relatively) small reduction in airway eosinophilia mediated by IL-5 blockade is insufficient to produce a therapeutic effect, or that the effects of anti-IgE therapy result from attenuation of multiple effector cells in the asthmatic inflammatory cascade and not solely a reduction in tissue eosinophils [35].

### 4.3. Bronchial Thermoplasty

Bronchial thermoplasty (BT) delivers thermal energy to the airway wall in a controlled manner to reduce excessive ASM [163]. The procedure has been well studied in severe persistent asthma that is not well controlled with inhaled corticosteroids and long-acting beta-agonists [164, 165]. Long-term followup of BT study patients supports the efficacy and safety of BT out to 5 years [166]. Lung function (FEV_1_ and FVC) remained stable over five years of followup. However, while studies have established small but significant improvements in PC_20_ doubling in patients undergoing BT when compared to controls for periods of up to 3 years after the procedure [166], there has in general been a lack of evidence demonstrating reduction in AHR. In the largest trial of bronchial thermoplasty to date [164], a subset of participants (100 treated with thermoplasty, 50 received sham bronchoscopy) underwent CT scans before and one year after treatment. Qualitative analysis of these images demonstrated no evidence of airway or parenchymal injury related to bronchial thermoplasty and an increase in bronchial wall thickening in those receiving sham bronchoscopy [167]. Therefore, thermoplasty may represent a mechanism by which smooth muscle can be abrogated resulting in the prevention of progressive remodeling in severe asthma.

## 5. Conclusions

There is now a substantial body of evidence documenting typical structural changes in the airways and lung parenchyma of asthmatic patients. These changes most likely contribute to the AHR and irreversible or partially reversible airflow obstruction seen in subgroups of asthmatic patients, especially those with more severe disease. The mechanisms responsible for these changes present viable therapeutic targets for the prevention and treatment of airway remodeling in asthma.

## References

[1] Anderson GP (2008). Endotyping asthma: new insights into key pathogenic mechanisms in a complex, heterogeneous disease. *The Lancet*.

[2] Grol MH, Gerritsen J, Vonk JM (1999). Risk factors for growth and decline of lung function in asthmatic individuals up to age 42 years: a 30-year follow-up study. *American Journal of Respiratory and Critical Care Medicine*.

[3] Lange P, Parner J, Vestbo J, Schnohr P, Jensen G (1998). A 15-year follow-up study of ventilatory function in adults with asthma. *New England Journal of Medicine*.

[4] Kelly WJW, Hudson I, Raven J, Phelan PD, Pain MCF, Olinsky A (1988). Childhood asthma and adult lung function. *American Review of Respiratory Disease*.

[5] Homer RJ, Elias JA (2005). Airway remodeling in asthma: therapeutic implications of mechanisms. *Physiology*.

[6] Carroll N, Elliot J, Morton A, James A (1993). The structure of large and small airways in nonfatal and fatal asthma. *American Review of Respiratory Disease*.

[7] James AL, Pare PD, Hogg JC (1989). The mechanics of airway narrowing in asthma. *American Review of Respiratory Disease*.

[8] Saetta M, Di Stefano A, Rosina C, Thiene G, Fabbri LM (1991). Quantitative structural analysis of peripheral airways and arteries in sudden fatal asthma. *American Review of Respiratory Disease*.

[9] Elias JA, Zhu Z, Chupp G, Homer RJ (1999). Airway remodeling in asthma. *Journal of Clinical Investigation*.

[10] Kuwano K, Bosken CH, Pare PD, Bai TR, Wiggs BR, Hogg JC (1993). Small airways dimensions in asthma and in chronic obstructive pulmonary disease. *American Review of Respiratory Disease*.

[11] James AJ, Stewart AG (1997). Relationship between airway wall thickness and airway hyperesponsiveness. *Airway Wall Remodeling in Asthma*.

[12] Awadh N, Müller NL, Park CS, Abboud RT, FitzGerald JM (1998). Airway wall thickness in patients with near fatal asthma and control groups: assessment with high resolution computed tomographic scanning. *Thorax*.

[13] Little SA, Sproule MW, Cowan MD (2002). High resolution computed tomographic assessment of airway wall thickness in chronic asthma: reproducibility and relationship with lung function and severity. *Thorax*.

[14] Niimi A, Matsumoto H, Amitani R (2000). Airway wall thickness in asthma assessed by computed tomography: relation to clinical indices. *American Journal of Respiratory and Critical Care Medicine*.

[15] Aikawa T, Shimura S, Sasaki H, Ebina M, Takishima T (1992). Marked goblet cell hyperplasia with mucus accumulation in the airways of patients who died of severe acute asthma attack. *Chest*.

[16] Li X, Wilson JW (1997). Increased vascularity of the bronchial mucosa in mild asthma. *American Journal of Respiratory and Critical Care Medicine*.

[17] Roche WR, Williams JH, Beasley R, Holgate ST (1989). Subepithelial fibrosis in the bronchi of asthmatics. *Lancet*.

[18] Castro M, Fain SB, Hoffman EA, Gierada DS, Erzurum SC, Wenzel S (2011). Lung imaging in asthmatic patients: the picture is clearer. *Journal of Allergy and Clinical Immunology*.

[19] Kasahara K, Shiba K, Ozawa T, Okuda K, Adachi M (2002). Correlation between the bronchial subepithelial layer and whole airway wall thickness in patients with asthma. *Thorax*.

[20] Aysola RS, Hoffman EA, Gierada D (2008). Airway remodeling measured by multidetector CT is increased in severe asthma and correlates with pathology. *Chest*.

[21] Pascual RM, Peters SP (2005). Airway remodeling contributes to the progressive loss of lung function in asthma: an overview. *Journal of Allergy and Clinical Immunology*.

[22] Holgate ST, Arshad HS, Roberts GC, Howarth PH, Thurner P, Davies DE (2010). A new look at the pathogenesis of asthma. *Clinical Science*.

[23] Cohn L, Elias JA, Chupp GL (2004). Asthma: mechanisms of disease persistence and progression. *Annual Review of Immunology*.

[24] Zhu Z, Homer RJ, Wang Z (1999). Pulmonary expression of interleukin-13 causes inflammation, mucus hypersecretion, subepithelial fibrosis, physiologic abnormalities, and eotaxin production. *Journal of Clinical Investigation*.

[25] Lee JJ, McGarry MP, Farmer SC (1997). Interleukin-5 expression in the lung epithelium of transgenic mice leads to pulmonary changes pathognomonic of asthma. *Journal of Experimental Medicine*.

[26] Jain-Vora S, Wert SE, Temann UA, Rankin JA, Whitsett JA (1997). Interleukin-4 alters epithelial cell differentiation and surfactant homeostasis in the postnatal mouse lung. *American Journal of Respiratory Cell and Molecular Biology*.

[27] Rankin JA, Picarella DE, Geba GP (1996). Phenotypic and physiologic characterization of transgenic mice expressing interleukin 4 in the lung: lymphocytic and eosinophilic inflammation without airway hyperreactivity. *Proceedings of the National Academy of Sciences of the United States of America*.

[28] Balzar S, Fajt ML, Comhair SAA (2011). Mast cell phenotype, location, and activation in severe asthma: data from the severe asthma research program. *American Journal of Respiratory and Critical Care Medicine*.

[29] Pearce N, Pekkanen J, Beasley R (1999). How much asthma is really attributable to atopy?. *Thorax*.

[30] Turato G, Barbato A, Baraldo S (2008). Nonatopic children with multitrigger wheezing have airway pathology comparable to atopic asthma. *American Journal of Respiratory and Critical Care Medicine*.

[31] The Lancet (2008). Asthma: still more questions than answers. *The Lancet*.

[32] Sumi Y, Hamid Q (2007). Airway remodeling in asthma. *Allergology International*.

[33] Holgate ST, Davies DE, Lackie PM, Wilson SJ, Puddicombe SM, Lordan JL (2000). Epithelial-mesenchymal interactions in the pathogenesis of asthma. *Journal of Allergy and Clinical Immunology*.

[34] Holgate ST (2007). Epithelium dysfunction in asthma. *Journal of Allergy and Clinical Immunology*.

[35] Holgate ST, Holloway J, Wilson S, Bucchieri F, Puddicombe S, Davies DE (2004). Epithelial-mesenchymal communication in the pathogenesis of chronic asthma. *Proceedings of the American Thoracic Society*.

[36] Patel AC, Morton JD, Kim EY (2006). Genetic segregation of airway disease traits despite redundancy of calcium-activated chloride channel family members. *Physiological Genomics*.

[37] Tyner JW, Kim EY, Ide K (2006). Blocking airway mucous cell metaplasia by inhibiting EGFR antiapoptosis and IL-13 transdifferentiation signals. *Journal of Clinical Investigation*.

[38] Grayson MH, Cheung D, Rohlfing MM (2007). Induction of high-affinity IgE receptor on lung dendritic cells during viral infection leads to mucous cell metaplasia. *Journal of Experimental Medicine*.

[39] Cheung DS, Ehlenbach SJ, Kitchens RT (2010). Cutting edge: CD49d+ neutrophils induce Fc*ε*RI expression on lung dendritic cells in a mouse model of postviral asthma. *Journal of Immunology*.

[40] Walter MJ, Morton JD, Kajiwara N, Agapov E, Holtzman MJ (2002). Viral induction of a chronic asthma phenotype and genetic segregation from the acute response. *Journal of Clinical Investigation*.

[41] Kim EY, Battaile JT, Patel AC (2008). Persistent activation of an innate immune response translates respiratory viral infection into chronic lung disease. *Nature Medicine*.

[42] Benoit LA, Holtzman MJ (2010). New immune pathways from chronic post-viral lung disease. *Annals of the New York Academy of Sciences*.

[43] Holtzman MJ, Patel DA, Zhang Y, Patel AC (2011). Host epithelial-viral interactions as cause and cure for asthma. *Current Opinion in Immunology*.

[44] Domachowske JB, Rosenberg HF (1999). Respiratory syncytial virus infection: immune response, immunopathogenesis, and treatment. *Clinical Microbiology Reviews*.

[45] Ahmed R, Morrison LA, Knipe DM, Knipe BN, Knipe DM, Howley PM (1996). Persistence of viruses. *Fields Virology*.

[46] Sigurs N (2001). Epidemiologic and clinical evidence of a respiratory syncytial virus-reactive airway disease link. *American Journal of Respiratory and Critical Care Medicine*.

[47] NAYLOR B (1962). The shedding of the mucosa of the bronchial tree in asthma. *Thorax*.

[48] Laitinen LA, Heino M, Laitinen A (1985). Damage of the airway epithelium and bronchial reactivity in patients with asthma. *American Review of Respiratory Disease*.

[49] Cohen L, Xueping E, Tarsi J (2007). Epithelial cell proliferation contributes to airway remodeling in severe asthma. *American Journal of Respiratory and Critical Care Medicine*.

[50] Ordoñez C, Ferrando R, Hyde DM, Wong HH, Fahy JV (2000). Epithelial desquamation in asthma: artifact or pathology?. *American Journal of Respiratory and Critical Care Medicine*.

[51] Jenkins HA, Cool C, Szefler SJ (2003). Histopathology of severe childhood asthma: a case series. *Chest*.

[52] Ordoñez CL, Khashayar R, Wong HH (2001). Mild and moderate asthma is associated with airway goblet cell hyperplasia and abnormalities in mucin gene expression. *American Journal of Respiratory and Critical Care Medicine*.

[53] Tagaya E, Tamaoki J (2007). Mechanisms of airway remodeling in asthma. *Allergology International*.

[54] Fahy JV (2001). Remodeling of the airway epithelium in asthma. *American Journal of Respiratory and Critical Care Medicine*.

[55] Cohn L, Tepper JS, Bottomly K (1998). Cutting edge: IL-4-independent induction of airway hyperresponsiveness by Th2, but not Th1, cells. *Journal of Immunology*.

[56] Kuperman DA, Huang X, Koth LL (2002). Direct effects of interleukin-13 on epithelial cells cause airway hyperreactivity and mucus overproduction in asthma. *Nature Medicine*.

[57] Amishima M, Munakata M, Nasuhara Y (1998). Expression of epidermal growth factor and epidermal growth factor receptor immunoreactivity in the asthmatic human airway. *American Journal of Respiratory and Critical Care Medicine*.

[58] Puddicombe SM, Polosa R, Richter A (2000). Involvement of the epidermal growth factor receptor in epithelial repair in asthma. *FASEB Journal*.

[59] Takeyama K, Fahy JV, Nadel JA (2001). Relationship of epidermal growth factor receptors to goblet cell production in human bronchi. *American Journal of Respiratory and Critical Care Medicine*.

[60] Demoly P, Simony-Lafontaine J, Chanez P (1994). Cell proliferation in the bronchial mucosa of asthmatics and chronic bronchitics. *American Journal of Respiratory and Critical Care Medicine*.

[61] Huber HL, Koessler KK (1922). The pathology of bronchial asthma. *Archives of Internal Medicine*.

[62] Homer RJ, Elias JA (2000). Consequences of long-term inflammation: airway remodeling. *Clinics in Chest Medicine*.

[63] Huang J, Olivenstein R, Taha R, Hamid Q, Ludwig M (1999). Enhanced proteoglycan deposition in the airway wall of atopic asthmatics. *American Journal of Respiratory and Critical Care Medicine*.

[64] Laitinen A, Altraja A, Kämpe M, Linden M, Virtanen I, Laitinen LA (1997). Tenascin is increased in airway basement membrane of asthmatics and decreased by an inhaled steroid. *American Journal of Respiratory and Critical Care Medicine*.

[65] Payne DNR, Rogers AV, Ädelroth E (2003). Early thickening of the reticular basement membrane in children with difficult asthma. *American Journal of Respiratory and Critical Care Medicine*.

[66] Boulet LP, Laviolette M, Turcotte H (1997). Bronchial subepithelial fibrosis correlates with airway responsiveness to methacholine. *Chest*.

[67] Chakir J (1996). Lower airways remodeling in nonasthmatic subjects with allergic rhinitis. Laboratory investigation. *Laboratory Investigation*.

[68] Chu HW, Halliday JL, Martin RJ, Leung DYM, Szefler SJ, Wenzel SE (1998). Collagen deposition in large airways may not differentiate severe asthma from milder forms of the disease. *American Journal of Respiratory and Critical Care Medicine*.

[69] Wenzel SE, Schwartz LB, Langmack EL (1999). Evidence that severe asthma can be divided pathologically into two inflammatory subtypes with distinct physiologic and clinical characteristics. *American Journal of Respiratory and Critical Care Medicine*.

[70] Benayoun L, Druilhe A, Dombret MC, Aubier M, Pretolani M (2003). Airway structural alterations selectively associated with severe asthma. *American Journal of Respiratory and Critical Care Medicine*.

[71] Chetta A, Foresi A, Del Donno M, Bertorelli G, Pesci A, Olivieri D (1997). Airways remodeling is a distinctive feature of asthma and is related to severity of disease. *Chest*.

[72] Cho SH, Seo JY, Choi DC (1996). Pathological changes according to the severity of asthma. *Clinical and Experimental Allergy*.

[73] Chun Geun Lee, Homer RJ, Zhu Z (2001). Interleukin-13 induces tissue fibrosis by selectively stimulating and activating transforming growth factor *β*1. *Journal of Experimental Medicine*.

[74] Batra V, Musani AI, Hastie AT (2004). Bronchoalveolar lavage fluid concentrations of transforming growth factor (TGF)-*β*1, TGF-*β*2, interleukin (IL)-4 and IL-13 after segmental allergen challenge and their effects on *α*-smooth muscle actin and collagen III synthesis by primary human lung fibroblasts. *Clinical and Experimental Allergy*.

[75] Branton MH, Kopp JB (1999). TGF-*β* and fibrosis. *Microbes and Infection*.

[76] Minshall EM, Leung DYM, Martin RJ (1997). Eosinophil-associated TGF-*β*1 mRNA expression and airways fibrosis in bronchial asthma. *American Journal of Respiratory Cell and Molecular Biology*.

[77] Ohno I, Nitta Y, Yamauchi K (1996). Transforming growth factor *β*1 (TGF*β*1) gene expression by eosinophils in asthmatic airway inflammation. *American Journal of Respiratory Cell and Molecular Biology*.

[78] Boulet LP, Belanger M, Carrier G (1995). Airway responsiveness and bronchial-wall thickness in asthma with or without fixed airflow obstruction. *American Journal of Respiratory and Critical Care Medicine*.

[79] Visse R, Nagase H (2003). Matrix metalloproteinases and tissue inhibitors of metalloproteinases: structure, function, and biochemistry. *Circulation Research*.

[80] Vignola AM, Riccobono L, Mirabella A (1998). Sputum metalloproteinase-9/tissue inhibitor of metalloproteinase-1 ratio correlates with airflow obstruction in asthma and chronic bronchitis. *American Journal of Respiratory and Critical Care Medicine*.

[81] Hoshino M, Nakamura Y, Sim J, Shimojo J, Isogai S (1998). Bronchial subepithelial fibrosis and expression of matrix metalloproteinase-9 in asthmatic airway inflammation. *Journal of Allergy and Clinical Immunology*.

[82] Mautino G, Henriquet C, Gougat C (1999). Increased expression of tissue inhibitor of metalloproteinase-1 and loss of correlation with matrix metalloproteinase-9 by macrophages in asthma. *Laboratory Investigation*.

[83] Kelly EA, Jarjour NN (2003). Role of matrix metalloproteinases in asthma. *Current Opinion in Pulmonary Medicine*.

[84] Hyytiäinen M, Penttinen C, Keski-Oja J (2004). Latent TGF-*β* binding proteins: extracellular matrix association and roles in TGF-*β* activation. *Critical Reviews in Clinical Laboratory Sciences*.

[85] Swartz MA, Tschumperlin DJ, Kamm RD, Drazen JM (2001). Mechanical stress is communicated between different cell types to elicit matrix remodeling. *Proceedings of the National Academy of Sciences of the United States of America*.

[86] Hastie AT, Kraft WK, Nyce KB (2002). Asthmatic epithelial cell proliferation and stimulation of collagen production: human asthmatic epithelial cells stimulate collagen type III production by human lung myofibroblasts after segmental allergen challenge. *American Journal of Respiratory and Critical Care Medicine*.

[87] Redington AE, Madden J, Frew AJ (1997). Transforming growth factor-*β*1 in asthma: measurement in bronchoalveolar lavage fluid. *American Journal of Respiratory and Critical Care Medicine*.

[88] Beqaj S, Jakkaraju S, Mattingly RR, Pan D, Schuger L (2002). High RhoA activity maintains the undifferentiated mesenchymal cell phenotype, whereas RhoA down-regulation by laminin-2 induces smooth muscle myogenesis. *Journal of Cell Biology*.

[89] Hirst SJ, Martin JG, Bonacci JV (2004). Proliferative aspects of airway smooth muscle. *Journal of Allergy and Clinical Immunology*.

[90] Schmidt M, Sun G, Stacey MA, Mori L, Mattoli S (2003). Identification of circulating fibrocytes as precursors of bronchial myofibroblasts in asthma. *Journal of Immunology*.

[91] Bergeron C, Al-Ramli W, Hamid Q (2009). Remodeling in asthma. *Proceedings of the American Thoracic Society*.

[92] Hossain S (1973). Quantitative measurement of bronchial muscle in men with asthma. *American Review of Respiratory Disease*.

[93] Freyer AM, Johnson SR, Hall IP (2001). Effects of growth factors and extracellular matrix on survival of human airway smooth muscle cells. *American Journal of Respiratory Cell and Molecular Biology*.

[94] Howarth PH, Knox AJ, Amrani Y, Tliba O, Panettieri RA, Johnson M (2004). Synthetic responses in airway smooth muscle. *Journal of Allergy and Clinical Immunology*.

[95] Page K, Li J, Wang Y, Kartha S, Pestell RG, Hershenson MB (2000). Regulation of cyclin D(1) expression and DNA synthesis by phosphatidylinositol 3-kinase in airway smooth muscle cells. *American Journal of Respiratory Cell and Molecular Biology*.

[96] Orsini MJ, Krymskaya VP, Eszterhas AJ, Benovic JL, Panettieri RA, Penn RB (1999). MAPK superfamily activation in human airway smooth muscle: mitogenesis requires prolonged p42/p44 activation. *American Journal of Physiology*.

[97] Madison JM (2003). Migration of airway smooth muscle cells. *American Journal of Respiratory Cell and Molecular Biology*.

[98] Hoshino M, Takahashi M, Aoike N (2001). Expression of vascular endothelial growth factor, basic fibroblast growth factor, and angiogenin immunoreactivity in asthmatic airways and its relationship to angiogenesis. *Journal of Allergy and Clinical Immunology*.

[99] Tanaka H, Yamada G, Saikai T (2003). Increased Airway Vascularity in Newly Diagnosed Asthma Using a High-magnification Bronchovideoscope. *American Journal of Respiratory and Critical Care Medicine*.

[100] Salvato G (2001). Quantitative and morphological analysis of the vascular bed in bronchial biopsy specimens from asthmatic and non-asthmatic subjects. *Thorax*.

[101] Orsida BE, Ward C, Li X (2001). Effect of a long-acting *β*2-agonist over three months on airway wall vascular remodeling in asthma. *American Journal of Respiratory and Critical Care Medicine*.

[102] Orsida BE, Li X, Hickey B, Thien F, Wilson JW, Walters EH (1999). Vascularity in asthmatic airways: relation to inhaled steroid dose. *Thorax*.

[103] Vrugt B, Wilson S, Bron A, Holgate ST, Djukanovic R, Aalbers R (2000). Bronchial angiogenesis in severe glucocorticoid-dependent asthma. *European Respiratory Journal*.

[104] Neufeld G, Cohen T, Gengrinovitch S, Poltorak Z (1999). Vascular endothelial growth factor (VEGF) and its receptors. *FASEB Journal*.

[105] Lee CG, Link H, Baluk P (2004). Vascular endothelial growth factor (VEGF) induces remodeling and enhances TH2-mediated sensitization and inflammation in the lung. *Nature Medicine*.

[106] Corne J, Chupp G, Lee CG (2000). IL-13 stimulates vascular endothelial cell growth factor and protects against hyperoxic acute lung injury. *Journal of Clinical Investigation*.

[107] Hoshino M, Nakamura Y, Hamid QA (2001). Gene expression of vascular endothelial growth factor and its receptors and angiogenesis in bronchial asthma. *Journal of Allergy and Clinical Immunology*.

[108] Yong Chul Lee (2001). Vascular endothelial growth factor in patients with acute asthma. *Journal of Allergy and Clinical Immunology*.

[109] Bai TR, Knight DA (2005). Structural changes in the airways in asthma: observations and consequences. *Clinical Science*.

[110] Siddiqui S, Martin JG (2008). Structural aspects of airway remodeling in asthma. *Current Allergy and Asthma Reports*.

[111] Lambert RK, Wiggs BR, Kuwano K, Hogg JC, Pare PD (1993). Functional significance of increased airway smooth muscle in asthma and COPD. *Journal of Applied Physiology*.

[112] Yanai M, Sekizawa K, Ohrui T, Sasaki H, Takishima T (1992). Site of airway obstruction in pulmonary disease: direct measurement of intrabronchial pressure. *Journal of Applied Physiology*.

[113] Wagner EM, Bleecker ER, Permutt S, Liu MC (1998). Direct assessment of small airways reactivity in human subjects. *American Journal of Respiratory and Critical Care Medicine*.

[114] Kaminsky DA, Wenzel SE, Carcano C, Gurka D, Feldsien D, Irvin CG (1997). Hyperpnea-induced changes in parenchymal lung mechanics in normal subjects and in asthmatics. *American Journal of Respiratory and Critical Care Medicine*.

[115] Winkler T, Venegas JG (2007). Complex airway behavior and paradoxical responses to bronchoprovocation. *Journal of Applied Physiology*.

[116] Lutchen KR, Gillis H (1997). Relationship between heterogeneous changes in airway morphometry and lung resistance and elastance. *Journal of Applied Physiology*.

[117] Brown RH, Herold CJ, Hirshman CA, Zerhouni EA, Mitzner W (1993). Individual airway constrictor response heterogeneity to histamine assessed by high-resolution computed tomography. *Journal of Applied Physiology*.

[118] Brown RH, Mitzner W (1998). The myth of maximal airway responsiveness in vivo. *Journal of Applied Physiology*.

[119] Brown RH, Croisille P, Mudge B, Diemer FB, Permutt S, Togias A (2000). Airway narrowing in healthy humans inhaling methacholine without deep inspirations demonstrated by HRCT. *American Journal of Respiratory and Critical Care Medicine*.

[120] Gillis HL, Lutchen KR (1999). Airway remodeling in asthma amplifies heterogeneities in smooth muscle shortening causing hyperresponsiveness. *Journal of Applied Physiology*.

[121] Lutchen KR, Jensen A, Atileh H (2001). Airway constriction pattern is a central component of asthma severity: the role of deep inspirations. *American Journal of Respiratory and Critical Care Medicine*.

[122] Martin JG, Duguet A, Eidelman DH (2000). The contribution of airway smooth muscle to airway narrowing and airway hyperresponsiveness in disease. *European Respiratory Journal*.

[123] Bjorck T, Gustafsson LE, Dahlen SE (1992). Isolated bronchi from asthmatics are hyperresponsive to adenosine, which apparently acts indirectly by liberation of leukotrienes and histamine. *American Review of Respiratory Disease*.

[124] Mitchell RW, Ruhlmann E, Magnussen H, Leff AR, Rabe KF (1994). Passive sensitization of human bronchi augments smooth muscle shortening velocity and capacity. *American Journal of Physiology*.

[125] Bai TR (1991). Abnormalities in airway smooth muscle in fatal asthma: a comparison between trachea and bronchus. *American Review of Respiratory Disease*.

[126] Whicker SD, Armour CL, Black JL (1988). Responsiveness of bronchial smooth muscle from asthmatic patients to relaxant and contractile agonists. *Pulmonary Pharmacology*.

[127] Thomson NC (1987). In vivo versus in vitro human airway responsiveness to different pharmacologic stimuli. *American Review of Respiratory Disease*.

[128] Léguillette R, Lauzon AM (2008). Molecular mechanics of smooth muscle contractile proteins in airway hyperresponsiveness and asthma. *Proceedings of the American Thoracic Society*.

[129] Léguillette R, Laviolette M, Bergeron C (2009). Myosin, transgelin, and myosin light chain kinase expression and function in asthma. *American Journal of Respiratory and Critical Care Medicine*.

[130] Ma X, Cheng Z, Kong H (2002). Changes in biophysical and biochemical properties of single bronchial smooth muscle cells from asthmatic subjects. *American Journal of Physiology - Lung Cellular and Molecular Physiology*.

[131] Duguet A, Biyah K, Minshall E (2000). Bronchial responsiveness among inbred mouse strains: role of airway smooth-muscle shortening velocity. *American Journal of Respiratory and Critical Care Medicine*.

[132] Wilson JW, Li X, Pain MCF (1993). The lack of distensibility of asthmatic airways. *American Review of Respiratory Disease*.

[133] Brackel HJL, Pedersen OF, Mulder PGH, Overbeek SE, Kerrebijn KF, Bogaard JM (2000). Central airways behave more stiffly during forced expiration in patients with asthma. *American Journal of Respiratory and Critical Care Medicine*.

[134] Ward C, Johns DP, Bish R (2001). Reduced airway distensibility, fixed airflow limitation, and airway wall remodeling in asthma. *American Journal of Respiratory and Critical Care Medicine*.

[135] Pitchford S, Page C (2003). Extracellular matrix composition influences the resistance of airway remodelling events towards glucocorticoid treatment. *British Journal of Pharmacology*.

[136] Fredberg JJ, Inouye D, Miller B (1997). Airway smooth muscle, tidal stretches, and dynamically determined contractile states. *American Journal of Respiratory and Critical Care Medicine*.

[137] Gunst SJ, Stropp JQ (1988). Pressure-volume and length-stress relationships in canine bronchi in vitro. *Journal of Applied Physiology*.

[138] Bai TR, Bates JHT, Brusasco V (2004). On the terminology for describing the length-force relationship and its changes in airway smooth muscle. *Journal of Applied Physiology*.

[139] McParland BE, Macklem PT, Paré PD (2003). Airway wall remodeling: friend or foe?. *Journal of Applied Physiology*.

[140] Fust A, Stephens NL (2005). Mechanical plasticity and contractile properties of airway smooth muscle. *Canadian Journal of Physiology and Pharmacology*.

[141] Bai TR, Cooper J, Koelmeyer T, Pare PD, Weir TD (2000). The effect of age and duration of disease on airway structure in fatal asthma. *American Journal of Respiratory and Critical Care Medicine*.

[142] Vanacker NJ, Palmans E, Kips JC, Pauwels RA (2001). Fluticasone inhibits but does not reverse allergen-induced structural airway changes. *American Journal of Respiratory and Critical Care Medicine*.

[143] Holgate ST (2009). Novel targets of therapy in asthma. *Current Opinion in Pulmonary Medicine*.

[144] Ward C, Pais M, Bish R (2002). Airway inflammation, basement membrane thickening and bronchial hyperresponsiveness in asthma. *Thorax*.

[145] Boulet LP, Turcotte H, Laviolette M (2000). Airway hyperresponsiveness, inflammation, and subepithelial collagen deposition in recently diagnosed versus long-standing mild asthma: influence of inhaled corticosteroids. *American Journal of Respiratory and Critical Care Medicine*.

[146] Tonascia J, Adkinson NF, Bender B (2000). Long-term effects of budesonide or nedocromil in children with asthma. *New England Journal of Medicine*.

[147] Shen HH, Ochkur SI, McGarry MP (2003). A causative relationship exists between eosinophils and the development of allergic pulmonary pathologies in the mouse. *Journal of Immunology*.

[148] Büttner C, Lun A, Splettstoesser T, Kunkel G, Renz H (2003). Monoclonal anti-interleukin-5 treatment suppresses eosinophil but not T-cell functions. *European Respiratory Journal*.

[149] Flood-Page PT, Menzies-Gow AN, Kay AB, Robinson DS (2003). Eosinophil’s role remains uncertain as anti-interleukin-5 only partially depletes numbers in asthmatic airway. *American Journal of Respiratory and Critical Care Medicine*.

[150] Kips JC, O’Connor BJ, Langley SJ (2003). Effect of SCH55700, a humanized anti-human interleukin-5 antibody, in severe persistent asthma: a pilot study. *American Journal of Respiratory and Critical Care Medicine*.

[151] Leckie MJ, Ten Brinke A, Khan J (2000). Effects of an interleukin-5 blocking monoclonal antibody on eosinophils, airway hyper-responsiveness, and the late asthmatic response. *Lancet*.

[152] Haldar P, Brightling CE, Hargadon B (2009). Mepolizumab and exacerbations of refractory eosinophilic asthma. *New England Journal of Medicine*.

[153] Nair P, Pizzichini MMM, Kjarsgaard M (2009). Mepolizumab for prednisone-dependent asthma with sputum eosinophilia. *New England Journal of Medicine*.

[154] Ayres JG, Higgins B, Chilvers ER, Ayre G, Blogg M, Fox H (2004). Efficacy and tolerability of anti-immunoglobulin E therapy with omalizumab in patients with poorly controlled (moderate-to-severe) allergic asthma. *Allergy*.

[155] Holgate ST, Chuchalin AG, Hébert J (2004). Efficacy and safety of a recombinant anti-immunoglobulin E antibody (omalizumab) in severe allergic asthma. *Clinical and Experimental Allergy*.

[156] Buhl R, Hanf G, Solèr M (2002). The anti-IgE antibody omalizumab improves asthma-related quality of life in patients with allergic asthma. *European Respiratory Journal*.

[157] Buhl R, Solèr M, Matz J (2002). Omalizumab provides long-term control in patients with moderate-to-severe allergic asthma. *European Respiratory Journal*.

[158] Busse W, Corren J, Lanier BQ (2001). Omalizumab, anti-IgE recombinant humanized monoclonal antibody, for the treatment of severe allergic asthma. *Journal of Allergy and Clinical Immunology*.

[159] Humbert M, Beasley R, Ayres J (2005). Benefits of omalizumab as add-on therapy in patients with severe persistent asthma who are inadequately controlled despite best available therapy (GINA 2002 step 4 treatment): INNOVATE. *Allergy*.

[160] Diane Lougheed M, Lemière C, Dell SD (2010). Canadian thoracic society asthma management continuum—2010 consensus summary for children six years of age and over, and adults. *Canadian Respiratory Journal*.

[161] Belliveau PP (2005). Omalizumab: a monoclonal anti-IgE antibody. *MedGenMed Medscape General Medicine*.

[162] Djukanović R, Wilson SJ, Kraft M (2004). Effects of treatment with anti-immunoglobulin E antibody omalizumab on airway inflammation in allergic asthma. *American Journal of Respiratory and Critical Care Medicine*.

[163] Miller JD, Cox G, Vincic L, Lombard CM, Loomas BE, Danek CJ (2005). A prospective feasibility study of bronchial thermoplasty in the human airway. *Chest*.

[164] Castro M, Rubin AS, Laviolette M (2010). Effectiveness and safety of bronchial thermoplasty in the treatment of severe asthma: a multicenter, randomized, double-blind, sham-controlled clinical trial. *American Journal of Respiratory and Critical Care Medicine*.

[165] Cox G, Thomson NC, Rubin AS (2007). Asthma control during the year after bronchial thermoplasty. *New England Journal of Medicine*.

[166] Thomson NC, Rubin AS, Niven RM (2011). Long-term (5 year) safety of bronchial thermoplasty: asthma Intervention Research (AIR) trial. *BMC Pulmonary Medicine*.

[167] Cox G (2009). Long term safety of bronchial thermoplasty (BT): 3 year data from multiple studies. *American Journal of Respiratory and Critical Care Medicine*.

